# Adsorption and oxidation of formaldehyde on a polycrystalline Pt film electrode: An in situ IR spectroscopy search for adsorbed reaction intermediates

**DOI:** 10.3762/bjnano.5.87

**Published:** 2014-05-30

**Authors:** Zenonas Jusys, R Jürgen Behm

**Affiliations:** 1 Institute of Surface Chemistry and Catalysis, Ulm University, Albert-Einstein-Allee 47, D-89081 Ulm, Germany

**Keywords:** electrocatalysis, formaldehyde adsorption, formyl intermediate, in situ spectro-electrochemistry, Pt

## Abstract

As part of a mechanistic study of the electrooxidation of C1 molecules we have systematically investigated the dissociative adsorption/oxidation of formaldehyde on a polycrystalline Pt film electrode under experimental conditions optimizing the chance for detecting weakly adsorbed reaction intermediates. Employing in situ IR spectroscopy in an attenuated total reflection configuration (ATR-FTIRS) with p-polarized IR radiation to further improve the signal-to-noise ratio, and using low reaction temperatures (3 °C) and deuterium substitution to slow down the reaction kinetics and to stabilize weakly adsorbed reaction intermediates, we could detect an IR absorption band at 1660 cm^−1^ characteristic for adsorbed formyl intermediates. This assignment is supported by an isotope shift in wave number. Effects of temperature, potential and deuterium substitution on the formation and disappearance of different adsorbed species (CO_ad_, adsorbed formate, adsorbed formyl), are monitored and quantified. Consequences on the mechanism for dissociative adsorption and oxidation of formaldehyde are discussed.

## Introduction

The electrooxidation of organic C1 molecules, in particular of methanol, has been one of the most important topics in electrocatalysis over the last decades, both from a fundamental aspect as a model reaction for the oxidation of more complex organic molecules and because of the potential application of these compounds as fuel in direct oxidation fuel cells [1]. In the meantime, it has been generally accepted that for all three C1 species, methanol, formaldehyde and formic acid, the reaction proceeds in a dual pathway mechanism (methanol oxidation [2–5], formaldehyde oxidation [6], formic acid oxidation [7–8]), with an indirect pathway proceeding via formation and subsequent oxidation of CO_ad_ and a direct pathway, where the reaction leads directly to CO_2_. The latter pathway allows the reaction to proceed already at potentials where CO_ad_ electrooxidation is still kinetically inhibited. In addition to complete oxidation to CO_2_, partial oxidation of methanol to formaldehyde and formic acid [9–12] and of formaldehyde to formic acid [6] have been identified as well as important contributions. These incomplete oxidation products may sensitively affect the reaction kinetics since they can i) be stepwise oxidized towards the final reaction product via a re-adsorption/further oxidation process [13–15], or ii) dissociatively adsorb to form CO_ad_, resulting in higher CO_ad_ coverages and hence enhanced surface poisoning [16]. Early in situ IR spectroscopy studies of C1 molecule oxidation had demonstrated the formation of adsorbed CO in the reaction, which was identified as reaction inhibiting side product [17–20]. More recently, Osawa and co-workers [21–26] and later also other groups [27–33] reported the formation of adsorbed, bridge-bonded formate species during oxidation of all three C1 molecules on Pt film electrodes. The role of the adsorbed formate as possible reaction intermediate in formic acid oxidation was addressed extensively in both experimental [21,25–28,34] and theoretical [35–38] studies.

So far, however, the elementary reaction steps and in particular the nature of the reaction intermediate(s) are still under intense debate (see, e.g., [39] and [40]). It is generally accepted that the reaction proceeds via a sequence of dehydrogenation and oxidation steps, as it had been beautifully described in the formal reaction scheme put forward by Bagotzky et al. [41]. Clear experimental evidence for partly dehydrogenated species, e.g., by spectroscopic observation, however, is still missing. In two early in situ IR spectroscopy studies on methanol oxidation, the authors reported the observation of weak bands at 1215 and 1270 cm^−1^, which they attributed to adsorbed –CH_x_OH [42] or –COH [43] intermediates, respectively. In later spectro-electrochemical studies, however, these features could not be reproduced, neither in an external reflection configuration (IRRAS), nor in highly sensitive surface enhanced IR spectroscopy measurements (SEIRAS) in an internal reflection configuration. Finally, the presence of hydrogen in the methanol adsorbate on an emersed polycrystalline Pt electrode was suggested from electrochemical thermal desorption mass spectrometry (ECTDMS) measurements based on the detection of carbon monoxide, hydrogen and traces of carbon dioxide during thermal desorption [44].

In a series of recent studies we have identified adsorbed acetyl with a characteristic band at about 1635 cm^−1^ or related species upon adsorption of higher alcohols on Pt electrodes (ethanol [45], ethylene glycol [46], 1-propanol [47], glycerol [48]) and demonstrated that they act as precursor for CO_ad_ and CO_2_ formation. Based on these findings, a similar reaction path, involving the formation of an adsorbed formyl intermediate, may also be expected for adsorption/oxidation of the C1 molecules. Adsorbed formyl species were indeed predicted as reaction intermediates in functional theory based theoretical studies of the interaction of methanol with Pt electrode surfaces, whereas in other studies adsorbed hydroxymethylidyne or adsorbed formaldehyde were suggested as adsorbed reaction intermediates [49–52]. This will be discussed in more detail below.

In the present contribution we want to further explore the formation of reaction intermediates during interaction of C1 molecules with Pt. Based on our results on the adsorption and oxidation of C2 and C3 molecules, where adsorbed acetyl-type species were most clearly visible at potentials up to 0.4 V (vs the reversible hydrogen electrode, RHE) and in the initial stages of the adsorption process, when surface blocking by strongly adsorbed CO_ad_ species is negligible or less pronounced, we followed the initial build-up of adsorbed species upon admission of formaldehyde molecule containing electrolyte to the electrode via in situ ATR-FTIR spectroscopy, optimizing the experimental conditions for the detection of weakly adsorbed reaction intermediates. Since signals of adsorbed acetyl-type species were more pronounced for the adsorption and oxidation of aldehydes than of the corresponding alcohols [45–48,53–54], we will focus here on the interaction of formaldehyde with Pt. Findings of a similar type study on the adsorption and oxidation of formic acid on Pt, where the reaction intermediates are different, will be published elsewhere. The experiments were performed in a thin-layer spectro-electrochemical flow cell [28] at constant potential (0.0–0.4 V), using a thin-film Pt electrode. In order to enhance the sensitivity towards weakly adsorbed reaction intermediates, the experiments were performed employing p-polarized IR radiation to improve the signal-to-noise ratio, and at low reaction temperatures, between room temperature and 3 °C. Low temperatures not only enhance the surface coverage of weakly adsorbed species, but may also increase the time window available for measurements at low CO_ad_ coverage by slowing down the decomposition or further oxidation of the adsorbed reaction intermediate. For the same reason, we also performed comparable measurements using deuterium labeled formaldehyde. In addition, this also provides information on the nature of the adsorbate. The rates of the CO_ad_ build-up were quantified and the kinetic H/D isotope effect in CO_ad_ formation was determined as a function of the electrode potential and temperature.

In the following, we will, after a brief description of the experimental procedures, present time resolved series of ATR-FTIR spectra recorded at different temperatures and potentials and using both H and D labeled compounds. We will compare the initial spectra to identify the adsorbed intermediates, followed by a discussion of the adsorption spectro-electrochemical transients. We will quantify the initial CO_ad_ formation rates from the respective molecules and their potential dependence to identify temperature effects and kinetic H/D isotope effect in the CO_ad_ formation reaction. Finally, the mechanistic implications of these findings for the C1 oxidation reaction will be discussed.

## Results and Discussion

### In situ ATR-FTIR spectra upon formaldehyde adsorption and oxidation

**Figure 1:**
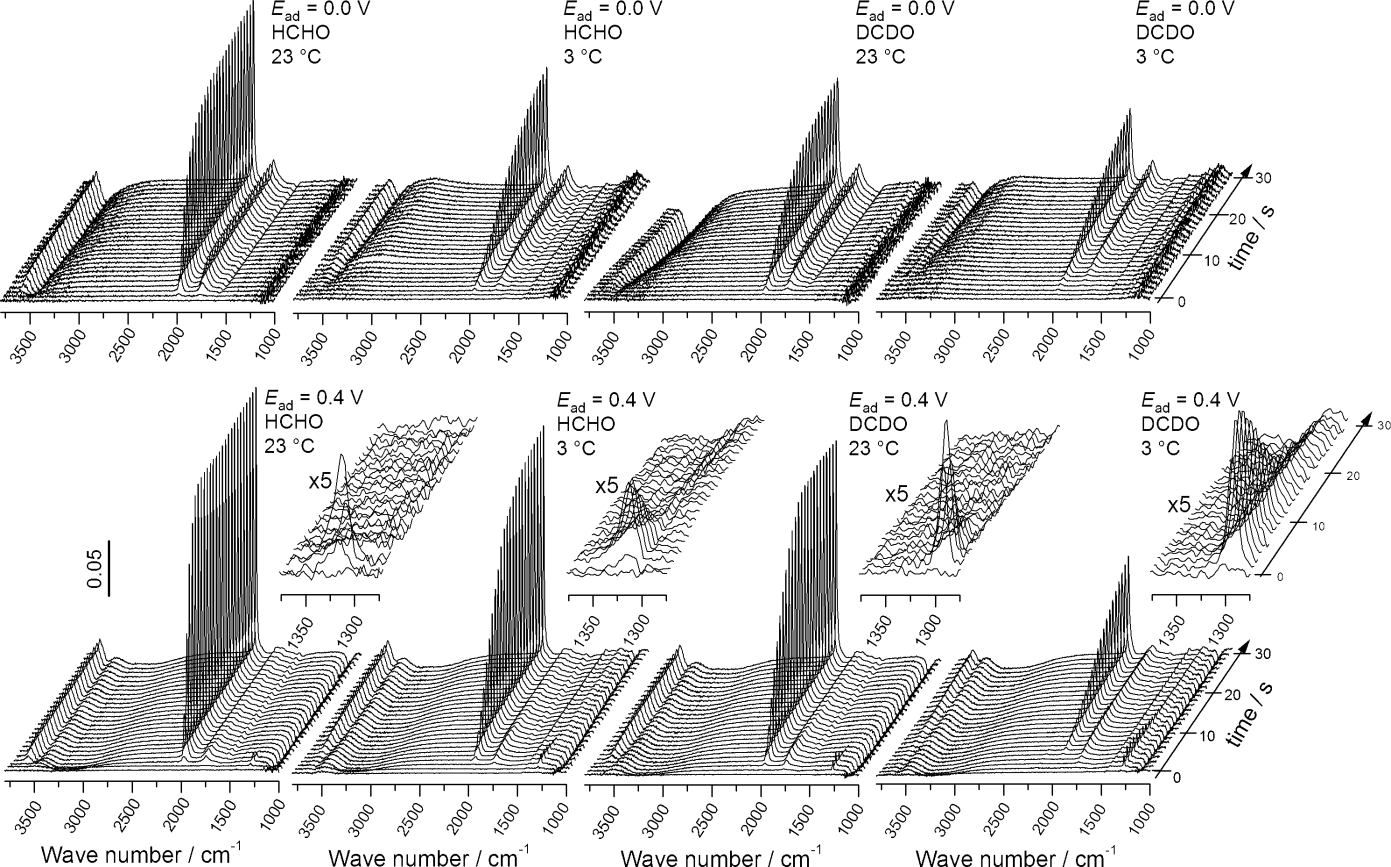
Temporal evolution of the ATR-FTIR spectra upon admission of 0.1 M H2- or D2-formaldehyde solution in 0.5 M HClO_4_ at 0.0 V (upper panel) or 0.4 V (lower panel) to a Pt film electrode at different temperatures (for notations see figure). Insets: five-fold magnified temporal evolution of adsorbed formate band.

**Figure 2:**
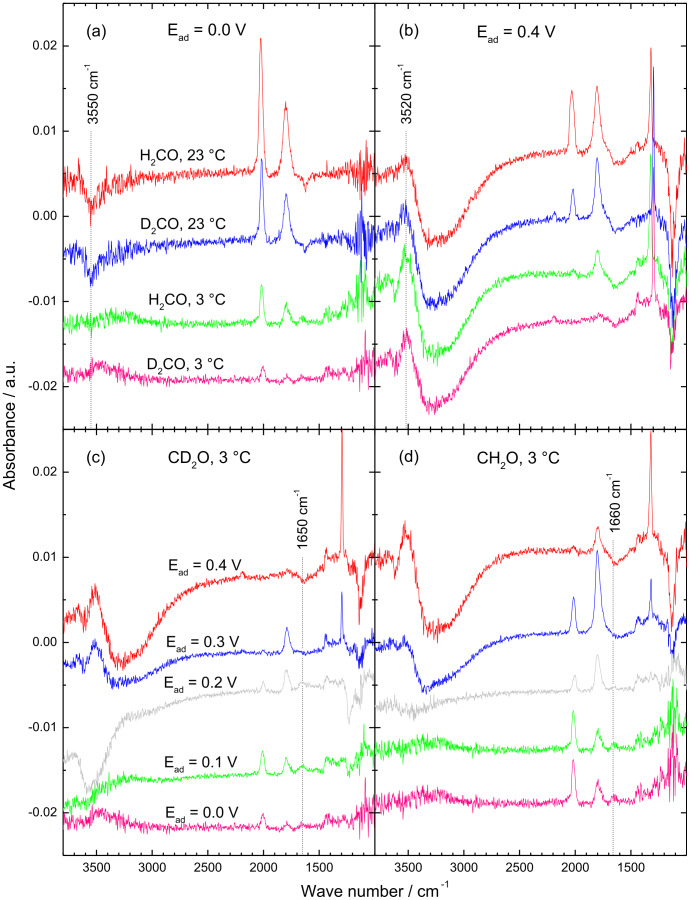
Initial ATR-FTIR spectra acquired ca. 2 s after admission of 0.1 M H2- or D2-formaldehyde solution in 0.5 M HClO_4_ to a film Pt electrode at: 0.0 V (a) and 0.4 V (b) at 23 and 3 °C; at 0.0, 0.1, 0.2, 0.3 and 0.4 V for 0.1 M D2- (c) or H2-formaldehyde (d) at 3 °C temperature (see figure for notations).

The temporal evolution of the ATR-FTIR spectra upon admission of either H2- or D2-formaldehyde to the Pt electrode surface at different temperatures (23 or 3 °C) and selected potentials (0.0 and 0.4 V) is shown in Figure 1. For better comparison, IR spectra acquired about 2 s after the admission of either H2- or D2-formaldehyde to the Pt electrode biased are plotted in Figure 2 for different potentials, isotopomers and temperatures. The temperature and isotope effects are illustrated in Figure 2a and Figure 2b for 0.0 V (2a) and 0.4 V (2b) adsorption potential, while the potential dependence of the initial spectra at 3 °C is depicted for D2- (c) and H2-formaldehyde (d) in Figure 2c and Figure 2d. Since the CO_ad_ coverage in the initial moments is still rather low, it is better possible to resolve bands of weakly adsorbed reaction intermediates, which otherwise may be overgrown by bands related to CO adsorption (see below).

The main characteristics of the spectra acquired at ambient temperature resemble those reported previously for ATR-FTIRS measurements in H_2_SO_4_ solution [23–24,32,55]. The bands at ca. 2010 cm^−1^ (at 0.0 V) are assigned to linearly bonded adsorbed CO (CO_L_), with the exact wave number depending both on the CO_ad_ coverage and the electrode potential [56]. The bands at ca. 1805 cm^−1^ result from multiply bonded CO (CO_M_). Bands related to displaced water, coadsorbed interfacial water or both, caused by the build-up of the CO adlayer, appear at wave numbers around 3500 cm^−1^ and at around 1620 cm^−1^ for the stretching and bending modes, respectively, in agreement with previous findings [21,57].

For adsorption at low potentials (0.0 to 0.3 V) and at low temperature, two weak broad positive bands appear at ca. 1420 and 1280 cm^−1^ with some residual intensity in between as well as a single broad band at around 1100 cm^−1^ (Figure 2c and Figure 2d). Bipolar bands at around 1425/1470, and around 1222/1245 cm^−1^ (positive/negative band) were reported previously for formaldehyde oxidation over a Pt(111) electrode [6]. Those authors tentatively assigned them to the symmetric deformation (scissoring) mode of a –CH_2_– (or –O–CH_2_) group for the former and to the C–O stretching mode of a –COH group for the latter band in a methylene glycolate adspecies, which is adsorbed via two oxygen atoms. Due to the rather low intensity of these bands and their broad shape it is not possible to resolve the expected shift in wave number for the isotopomers (Figure 2). Note that these bands were not observed for room temperature adsorption (Figure 2a), although methylene glycol is equally present in the solution (see discussion below). Possible reasons herefore will be discussed below. At 0.3 V, and more strongly at 0.4 V, a pronounced negative band at ca. 1110 cm^−1^ developed, which is associated with the displacement of adsorbed perchlorate species [58]. It should be noted that adsorption of perchlorate was verified also by CO displacements transients, which showed a positive current at 0.1 and 0.2 V (*H*_upd_ displacement) and below, but a negative current at 0.3 and 0.4 V (adsorbed perchlorate displacement) (see Supporting Information File 1, Figure S1).

A distinct band at ca. 1320 cm^−1^ , which appears at 0.3 V and in particular at 0.4 V, has been assigned to the symmetric O–C–O vibration of a bridge bonded adsorbed formate [23–24,32,55]. This assignment is further supported by the corresponding shift of the band from 1320 to 1295 cm^−1^ when using deuterated formaldehyde (see the magnified insets in Figure 1).

A closer look at the initial IR spectra acquired at low adsorption potential (0.0 to 0.2 V, Figure 2c and Figure 2d) resolves a band at ca. 1650 cm^−1^ for D2-formaldehyde and at around 1660 cm^−1^ for H2-formaldehyde adsorption at 3 °C, which could be assigned to an adsorbed formyl species. A possible assignment of this band to water coadsorbed with CO_ad_ is unlikely due to the negligible CO_ad_ coverage in the initial stages of the adsorption process (see below). The assignment to adsorbed formyl is further supported by the red shift of the band by ca. 10 cm^−1^ upon deuteration of formaldehyde. This very weak band, whose intensity is close to the detection limit of the spectra, appeared reproducibly in a number of different experiments, both at 0.1 and 0.2 V adsorption potential, verifying that it is indeed due to absorption and not reflecting noise in the spectra.

The adsorbed formyl band is slightly less pronounced for adsorption of D2- than for H2-formaldehyde (Figure 2c and Figure 2d), which may reflect a kinetic H/D isotope effect (see section ‘Formaldehyde adsorption: CO_ad_ formation rate and the kinetic H/D isotope effect’ for kinetic isotope effects in the build-up of CO_ad_), but the differences are in the limits of the detection and shall therefore not be discussed in more detail at this point. For increasing CO_ad_ coverage, which is rapidly reached for the fast dehydrogenation at ambient temperature (Figure 2a), this peak can not longer be resolved because of its overlap with a negative peak evolving at rather similar wave number. The latter is most likely related to the displacement of water from the surface by CO adsorption (bending mode of displaced water). Finally, in the high wave number region, we find a weak broad negative band at around 3550 cm^−1^, whose appearance seems to be correlated with the formation of CO_ad_. Therefore, it is associated with the O–H stretching mode of displaced interface water due to CO adsorption (see below and [58]).

The observation of an adsorbed formyl species agrees perfectly with the clear identification of adsorbed acetyl-type species, with a characteristic band at 1635 cm^−1^, in a series of recent ATR-FTIR spectroscopy studies for adsorption of higher alcohols and aldehydes. [46–48,59–61]. In those studies, the barrier for C–C bond breaking stabilizes these acetyl-type adspecies against decomposition to CO_ad_, which allows to reach higher coverages and band intensities of these species. We could clearly demonstrate, by using deuterium [46,59] or ^13^C labeling [59,61], that the band is indeed due to adsorbed acetyl species and not caused by water coadsorbed with CO_ad_. In fact, even the red-shift upon deuteration of ca. 10 cm^−1^ obeserved in the present measurements agrees reasonably well with that obtained for deuterium labeled adsorbed acetyl species [46,59]. In the present case, the more facile C–H bond breaking leads to faster decomposition of the adsorbed formyl species, equivalent to a lower steady-state coverage. Overall, it was only possible to detect this species by combination of highly sensitive spectroscopy (SEIRAS using p-polarized light) and use of reaction conditions which slow down the rate for C–H bond breaking and the coverage of coadsorbed CO (low temperature, CO_ad_ free electrode at the initial stage of the adsorption transient = low CO_ad_ coverage).

The fact that observation and identification of adsorbed formyl species was possible only at low temperature and low potentials (Figure 2a and Figure 2c) implies that these intermediate species are highly reactive towards further dehydrogenation to the final stable state (CO_ad_). The decomposition to CO_ad_ is obviously slowed down by lowering the reaction temperature, but apparently also by the presence of a high coverage adsorbed hydrogen adlayer at low potentials (0.0 V). The latter may stabilize the adsorbed intermediate by site blocking, leaving no empty sites for CHO_ad_ decomposition [62]. The comparable intensities in CHO_ad_ and CDO_ad_ in combination with the slower build-up of CO_ad_ from the D2-formaldehyde (see section ‘Formaldehyde adsorption: CO_ad_ formation rate and the kinetic H/D isotope effect’) can be understood if both adsorbed formyl formation and decomposition are slowed down upon deuteration. On an absolute scale, however, the intensity of this band and hence also the coverage of this species are still very low.

Adsorbed formyl species were indeed predicted as reaction intermediates in density functional theory based studies of the interaction of methanol with a Pt(111) surface [50]. The importance of water in the initial steps of dehydrogenation of methanol over Pt(111) via polarization of the hydroxyl due to hydrogen bond formation with a neighboring water molecule was addressed in [63]. This favors the cleavage of the C–H bond upon adsorption in a concerted step, together with the O–H hydrogen transfer to a water molecule, which finally results in an HCHO_ad_ species. Density functional theory based calculations of the energy of dehydrogenation over solvated platinum surfaces were used to approximate the potential-dependent methanol dehydrogenation pathways over different low index Pt electrode surfaces [49]. These calculations revealed pronounced structural effects, in agreement with experimental findings. For Pt(111), they suggested the coexistence of two pathways, where the indirect pathway proceeds via formation of stable CO_ad_, via an initial exothermic C–H cleavage step to adsorbed hydroxymethyl, which occurs over a wide potential range, and its subsequent exothermic dehydrogenation steps to form CO_ad_. Another pathway (‘incomplete dehydrogenation’) was predicted to proceed via an initial O–H cleavage step to form adsorbed methoxy (which is expected to be competitive to C–H cleavage at quite positive potentials), followed by exothermic C–H cleavage to form adsorbed formaldehyde, which can subsequently desorb into solution. Similar type calculations, including water molecules and the electrode potential, implied that methanol dehydrogenation to CO_ad_ via a hydroxymethyl intermediate (initial C–H bond dissociation) is the lowest energy path, whereas the formation of a formaldehyde intermediate (initial C–H bond dissociation) is a minority pathway on a Pt(111) surface at 0.5 V (NHE) [51]. A recent detailed theoretical study on the stability, configuration and interconversion of formyl (CHO) and hydroxymethylidyne (COH) adsorbed on Pt(111) under a water bilayer suggested that CHO_ad_ is the only (meta-)stable form under these conditions, while the COH_ad_ conﬁguration dissociates easily to CO_ad_ + H [52]. For CHO_ad_ on a bridge site under a water bilayer, only a single C–O bond vibration was calculated at wave numbers of 1250 cm^−1^ [52].

For an adsorption potential of 0.4 V (Figure 2b), the initial IR spectra exhibit distinct differences compared to the spectra recorded at lower potentials (0.0–0.2 V). This includes significantly less intense CO_ad_ related bands, a sharp positive band at around 1320 cm^−1^, and a negative band at 1100 cm^−1^, where the latter two are attributed (see discussion above) to bridge-bonded adsorbed formate and to displaced adsorbed perchlorate species, respectively. In the high wave number region, an apparently bipolar feature appears, with a pronounced broad negative feature in the range from ca. 2500 to 3500 cm^−1^ and a positive component developed at about 3520 cm^−1^ (Figure 2b). These two features are distinctly different from the weak negative band formed at lower potentials at 3550 cm^−1^ (Figure 2a). According to Osawa et al., the new bipolar feature can be explained by a positive band centered at 3550 cm^−1^ (water coadsorbed with CO_ad_) superimposed on a broad negative band of the displaced water, which ranges from ca. 3700 to 2500 cm^−1^ [58]. For the present spectra, the positive peak may be due to water coadsorbed with adsorbed formates, since in the early stages (Figure 2b) the CO_ad_ coverages are negligible. This latter idea is supported by the fact that the spectral characteristics in this region are clearly different from those developed upon adsorption of CO dissolved in the solution, as found from comparison with the initial IR spectrum acquired upon adsorption of dissolved CO (see Supporting Information File 1, Figure S2). It should be noted that the wave number of this band (3520 cm^−1^) is close to the value of around 3635 cm^−1^ predicted (calculated) for the OH stretching mode of hydroxymethylidyne adsorbed on Pt(111) [52].

At this potential, there is no indication of any band at ca. 1660 cm^−1^ related to adsorbed formyl species (Figure 2a), despite of the much lower CO_ad_ coverage at short exposure times (Figure 2b, 2c and 2d). In this case, however, the rapid formation of adsorbed formates may lead to a negative band in this spectral range, due to displacement of interfacial water, which makes it impossible to resolve a possibly existent peak of adsorbed formyl in the early stages of the adsorption transient, despite of a very low CO_ad_ coverage. Hence, from the present data we can not decide, whether the missing band at 1660 cm^−1^ reflects the absence of adsorbed formyl species under these adsorption conditions or whether their signal is just overcompensated by the negative band of the displaced interfacial water.

**Figure 3:**
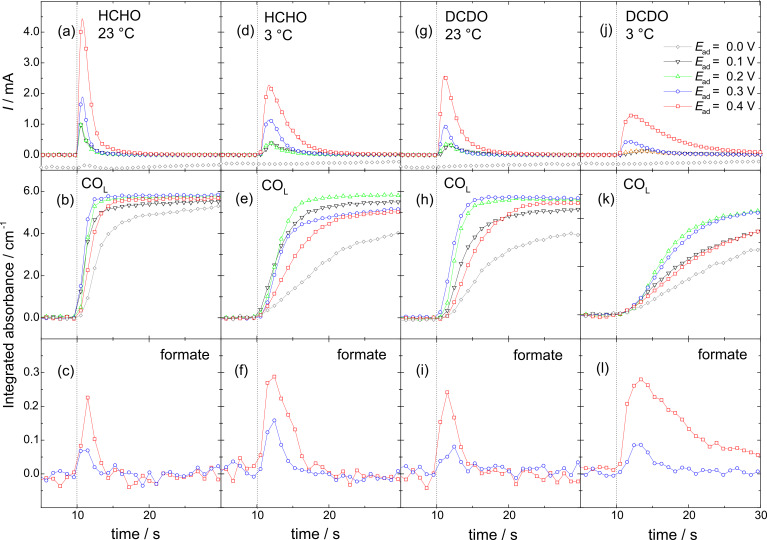
Transients of Faradaic current (upper panel) and integrated intensities of linearly bonded CO_ad_ (middle panel) and adsorbed bridge-bonded formate (for 0.3 and 0.4 V only) bands upon admission of 0.1 M H2- or D2-formaldehyde solution in 0.5 M HClO_4_ to a Pt film electrode biased at 0.0, 0.1, 0.2, 0.3 and 0.4 V at 23 or 3 °C temperature (see figure for notations).

Additional mechanistic insight comes from the time and potential dependent appearance and disappearance of the different features in these sequences of spectra. The transient appearance of the adsorbed formate band during the initial stages of formaldehyde admission to the Pt electrode surface at 0.3 V and 0.4 V (see the magnified insets in Figure 1 and transients in Figure 3) clearly indicates formaldehyde oxidation to formic acid under these conditions. Interestingly, the time span during which adsorbed formate is present on the surface correlates with the rate of CO_ad_ formation, with a longer presence of the formate band at a smaller CO_ad_ formation rate. The latter is reduced both by a lower adsorption temperature and/or by using deuterated formaldehyde. For the adsorption of D2-formaldehyde at 3 °C the adsorbed formate band exists over more than 10 seconds, whereas for H2-formaldehyde at 23 °C it instantaneously appears and vanishes within 1–2 seconds, together with a much faster CO_ad_ build-up. The transient appearance of adsorbed formate species is most easily explained by a mechanism where adsorbed CO increasingly blocks the surface for adsorption of the less strongly adsorbed formates. In agreement with that mechanism, the adsorbed formate band has essentially disappeared when a critical CO_ad_ coverage is reached, which is around 60% of the CO_ad_ saturation coverage independent of temperature and isotopomer.

The oxidation of formaldehyde to formic acid requires the addition of oxygen from the dissociative electrosorption of water, which, in contrast to Pt(100) [64], can essentially be ruled out on a polycrystalline Pt electrode at this low potential [65]. The absence of reactive OH_ad_ species on the electrode surface at these potentials can also be concluded from the constant CO_ad_ coverage after the adsorption transients, as evidenced by the unchanged CO_ad_ band intensity and wave number after switching back to the supporting electrolyte, between adsorption transients and subsequent CO_ad_ stripping experiments. Alternatively, one could envision a reaction pathway proceeding via adsorption of methylene glycol species, which are formed by hydration of formaldehyde in the solution phase [66–67]. Methylene glycol can be oxidized to adsorbed formates (formic acid) via a dehydrogenation step [6], without the need for OH_ad_ species. It is well known for methanol [4] and a series of higher alcohols that their dissociative adsorption on Pt surfaces is hindered by *H*_upd_ [45–48,60–61]. Correspondingly, one would expect that adsorption of methylene glycol is also inhibited by *H*_upd_, blocking also oxidation of methylene glycol to formic acid at high *H*_upd_ coverages (low potentials). This fully agrees with the experimental findings, where the adsorbed formate band is absent for formaldehyde adsorption at <0.3 V (Figure 1).

The formic acid formed upon adsorption and oxidation of the hydrated form of formaldehyde can be further oxidized to CO_2_. This was demonstrated by the transient CO_2_ formation upon adsorption of formaldehyde on a Pt electrode at ambient temperature at potentials of 0.3 and 0.4 V [32], where CO_2_ formation via an indirect pathway can be neglected. On the other hand, the strongly adsorbing carbonyl functional group of non-hydrated formaldehyde enables the dissociative adsorption to form CO_ad_ even at low potentials, e.g., via displacement of *H*_upd_. At 0.4 V, where there is no more *H*_upd_ blocking, methylene glycol can also dissociatively adsorb to form CO_ad_, via dissociation of two C–H bonds, assuming that it behaves similarly as other alcohols [45–48,60–61]. In addition, however, it could be oxidized to formic acid, which requires dissociation of a single C–H bond only, in an apparently facile dehydrogenation step.

Similar trends were also reported for the competing adsorption of formaldehyde and methanol using mixtures of carbon-labelled formaldehyde and methanol, which revealed a prevailing CO_ad_ formation from formaldehyde at low potentials (*H*_upd_ region), whereas in the double-layer region methanol was the dominant source for CO_ad_ formation [16]. Likewise, combining alcohol and aldehyde functional groups at different carbon atoms in a single molecule, in glycoladehyde, we obtained facile CO_ad_ formation at both low and high potential [68].

Overall, our data strongly support the idea that for understanding the interaction of formaldehyde with Pt electrodes, the coexistence of both the non-hydrated and hydrated form of formaldehyde in the bulk solution has to be considered. This is most clearly evident from the different trends in the potential dependence in formic acid (CO_2_ formation) and in CO_ad_ formation, which was discussed above. In that case, the (possible) absence of the band related to adsorbed formyl, the lower CO_ad_ coverage, and the transient appearance of adsorbed formates at 0.3 V and more pronounced at 0.4 V in combination may be explained by a simple mechanism, where dehydrogenation of formaldehyde to form CO_ad_, which prevails at low potentials and proceeds via adsorbed formyl, is partly replaced by oxidation of the hydrated form of formaldehyde (methylene glycol) to formic acid at 0.3 and 0.4 V. The reaction proceeds until it is stopped by CO_ad_ development.

Further mechanistic insight comes from the absence of isotope mixing in adsorbed formates resulting from adsorption of D2-formaldehyde. If desorption of adsorbed formyl species were possible via re-hydrogenation and subsequent desorption of the resulting adsorbed formaldehyde, re-hydrogenation of CDO_ad_ by protons (from water) should result in the formation of a mixed D1H1-formaldehyde. Its subsequent hydration to D1H1-methylene glycol and re-adsorption and oxidation to formic acid would result in a mixture of both D1- and H1-formate species. Within the detection limits, this does not seem to be the case (see the magnified insets in Figure 1, and Figure 2b, where only a single band of D1-formate is resolved). Therefore, desorption of the formyl intermediate as formaldehyde (as a possible reason for the absence of the band at 1650 cm^−1^ for D2-formaldehyde adsorption at 0.3 (0.4) V and 3 °C in Figure 2c) is unlikely because of the absence of an adsorbed H1-formate isotopomer, at least at significant rates.

### Formaldehyde adsorption and oxidation transients

The initial Faradaic current transients (upper panel) as well as the temporal evolution of the integral absorbances of linearly bonded CO_ad_ (CO_L_, middle panel) and of bridge-bonded formate (bottom panel) obtained upon the admission of H2- (Figure 3a–f) or D2- (Figure 3g–l) formaldehyde to the Pt electrode (at about 10 s) are plotted in Figure 3. The electrode was biased at constant potentials of 0.0, 0.1, 0.2, 0.3 and 0.4 V, the data were recorded at room temperature (Figure 3a–c and Figure 3g–i) and 3 °C (Figure 3d–f and Figure 3j–l). The integral absorbances were evaluated from sequences of IR spectra, which were shown for representative potentials of 0.0 and 0.4 V in Figure 1.

In general, the transients are similar to those recorded for formaldehyde adsorption in sulfuric acid solution at ambient temperature [16,32,55]. For adsorption at 0.0 V, the Faradaic current is governed by contributions from hydrogen evolution. The latter slightly decays with time, which can be explained by a build-up of CO_ad_ from dissociative adsorption of formaldehyde (see below). For potentials between 0.1 and 0.4 V, the Faradaic current increases steeply upon the admission of formaldehyde, passes a pronounced maximum and then decays in an approximately exponential form to zero. The Faradaic current peaks are highest and narrowest for H2-formaldehyde adsorption at ambient temperature. Both the temperature decrease and/or deuteration lead to lower maxima and peak broadening. The peak shape of the Faradaic current transients, specifically the decay after passing the maximum, indicates an increasing surface poisoning, most likely by the CO_ad_ resulting from the dissociative adsorption of formaldehyde. This is confirmed by the IR spectroscopy data (see CO_L_ transients below). The data presented so far clearly indicate that the Faradaic current peak results from at least three contributions: i) displacement of *H*_upd_ or adsorbed ClO_4_ species, depending on the adsorption potential, ii) the formation of CO_ad_ from adsorbed formaldehyde or methylene glycol, and iii) oxidation of adsorbed formaldehyde (methylene glycol) to adsorbed formates or formic acid and eventually to CO_2_. Formation of CO_ad_ from adsorbed formaldehyde will release two electrons upon formation of one CO_ad_ molecule, displacement of *H*_upd_ or adsorbed ClO_4_ will release (consume) one electron per atom (molecule), oxidation to formate or formic acid will release two electrons per molecule, and oxidation to CO_2_ four electrons per molecule. Because of the largely unknown product yields, in particular the yields of formic acid and CO_2_ are unknown in these experiments, it is not possible to identify and quantify the contributions from the different product formation rates, in particular at 0.3 V and higher, where formate, formic acid, and CO_2_ formation contribute increasingly. Nevertheless, due to the accumulation of reaction inhibiting CO_ad_, which can not be oxidized at these potentials (≤0.4 V), the Faradaic current eventually decreases to zero.

The integrated intensities of the CO_L_ band plotted in the middle row of Figure 3 show a fast increase upon admission of the formaldehyde containing solution to the Pt film electrode, indicating an instantaneous onset of dissociative formaldehyde adsorption. For H2-formaldehyde adsorption at room temperature (Figure 3b), they approach saturation within a few seconds, except for 0.4 V where it takes a bit longer, whereas for the lower temperature and/or deuteration of formaldehyde the CO_ad_ build-up is slower, so that saturation may not be reached during the time of the transient (middle panel of Figure 3). Looking at the potential dependence, CO_ad_ formation seems to be fastest at 0.1–0.3 V, and is somewhat slower for higher and lower potentials. Importantly, the rate of the CO_ad_ development is clearly correlated with the width of the Faradaic current transients, which will be discussed below. It should be noted that the CO_ad_ formation due to dissociative adsorption of formaldehyde at 0.0 V decreases the current for hydrogen evolution only slightly (see above). Apparently, an efficient hydrogen evolution is possible even on largely CO_ad_ blocked electrode [69].

The integrated intensities of the bridge bonded adsorbed formate band are plotted in the lower panel of Figure 3 for 0.3 and 0.4 V adsorption potentials (for lower potentials the formate band is not resolved and therefore they are not included). As discussed before (see previous section), the transient appearance of the adsorbed formate band at these low potentials supports a mechanism where formaldehyde oxidation to formic acid proceeds via the hydrated form of formaldehyde (methylene glycol) and its interaction with the initially adsorbate-free electrode. At lower potentials, the electrode surface is largely blocked by *H*_upd_, which inhibits the adsorption of alcohols [45–48,60–61] and thus the adsorption of methylene glycol.

The integrated absorbance of adsorbed formate (at adsorption potentials of 0.3 and 0.4 V) develops instantaneously upon exposure of the electrode to the reactant (Figure 3, bottom panel). After passing through a maximum, it decreases again until reaching the background level. As mentioned above, the duration of the adsorbed formate appearance is strictly correlated with the initial rate for CO_ad_ formation. It is shortest for the fastest CO_ad_ build-up (H2-formaldehyde adsorption at 23 °C, Figure 3a–c) and longest for the slowest build-up of CO_ad_ (D2-formaldehyde adsorption at 3 °C, Figure 3j–l). The transient appearance of the adsorbed formate species is typical of a self-poisoning behavior, in this case by adsorbed CO. which can displace the rather weakly bonded adsorbed formate species (see also the displacement of weakly adsorbed perchlorate indicated by the negative band at 0.3 and 0.4 V adsorption potential, Figure 1 and Figure 2b–d) and block the sites required for the further dehydrogenation of methylene glycol to formic acid [70]. Therefore, the adsorbed formate species should be considered as an indicator of methylene glycol oxidation to formic acid rather than as an active intermediate in formaldehyde oxidation to CO_2_, as had been suggested earlier [23–24]. The active intermediate in this case are adsorbed methylene glycolates. The oxidation of methylene glycol to formic acid is responsible for the ongoing Faradaic current during the build-up of CO_ad_ at 0.3 and in particular at 0.4 V. Accordingly, the total charge in the Faradaic current transient is highest for the adsorption of D2-formaldehyde at low temperature at 0.4 V (see Figure 3j–l), where the build-up of CO_ad_ is slowest.

### Formaldehyde adsorption: CO_ad_ formation rate and the kinetic H/D isotope effect

**Figure 4:**
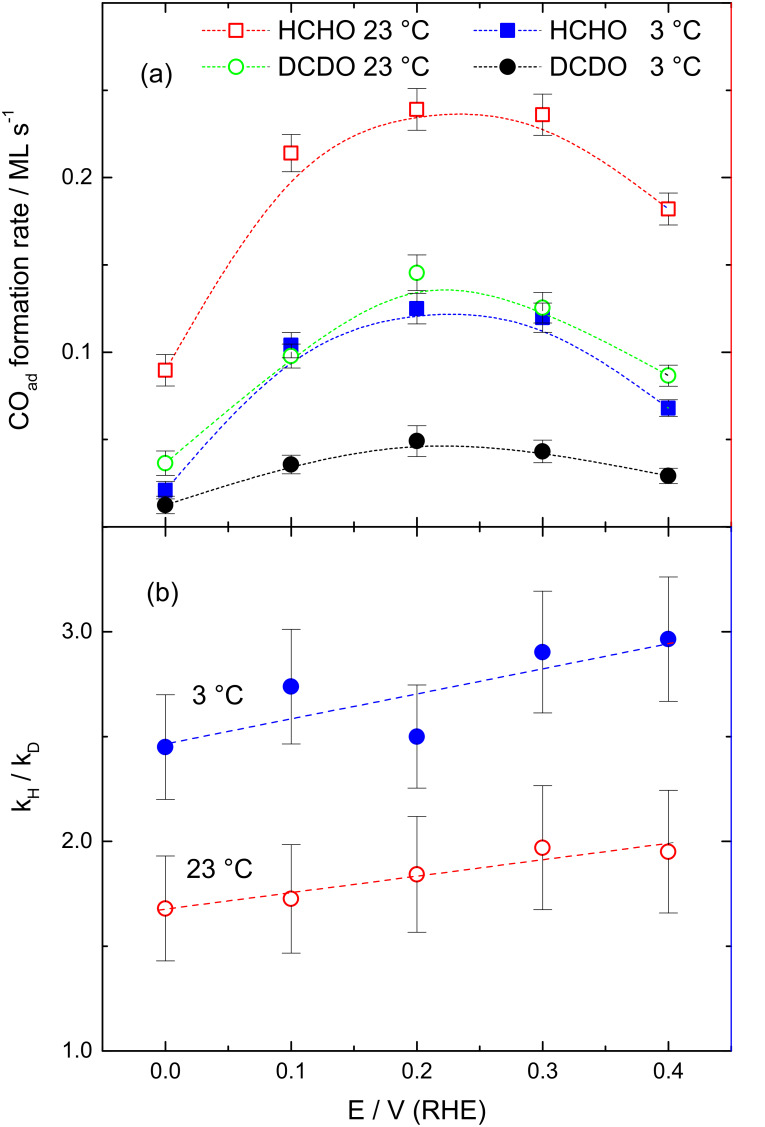
Initial CO_ad_ formation rates (a) and kinetic isotope effects for the CO_ad_ formation (b) upon admission of 0.1 M H2- or D2-formaldehyde to a Pt film electrode in 0.5 M HClO_4_ at different potentials and temperatures (see figure for notations). Lines are included to guide the eye.

Figure 4 shows the potential dependence of i) the initial rates of the CO_ad_ build-up upon admission of the reacting molecule to the electrode surface (a), and of ii) the kinetic H/D isotope effect (b) found as the ratio of the initial rates for CO_ad_ build-up from the corresponding isotopomers H2- and D2-formaldehyde at 23 and 3 °C. The initial CO_ad_ formation rates were determined from the CO_L_ intensity increase (see Experimental section). In general, the rates for CO_ad_ formation from the dissociative adsorption of formaldehyde show a substantial effect of the adsorption potential, with lower values at 0.0 and 0.4 V, and higher values in the range 0.1–0.3 V. Furthermore, they decrease to about half upon lowering the temperature to 3 °C or upon deuteration of the C–H bond (Figure 4a). The ability of formaldehyde to dissociatively adsorb even on a largely *H*_upd_ covered Pt surface is related to the strong affinity of the carbonyl group (aldehyde function) to interact with metal surfaces, which was also found for a number of higher aldehydes [45,47–48,71]. Only at very high coverages of *H*_upd_, as reached at 0.0 V and bulk evolution of H_2_, the rate for dissociative formaldehyde decreases significantly. The metal–carbonyl interaction is sufficiently strong to displace reversibly adsorbed *H*_upd_ [45,47–48,68,72], which allows dehydrogenation of adsorbed formaldehyde molecules to CO_ad_ also on *H*_upd_ covered surfaces. The less pronounced decrease in the initial CO_ad_ formation rate at 0.4 V could be interpreted as result of a competing oxidation of hydrated formaldehyde (methylene glycol) to formic acid as discussed above, thereby lowering the CO_ad_ formation rate (from adsorbed formaldehyde).

The decrease in the CO_ad_ formation rate upon formaldehyde dehydrogenation with temperature indicates a thermal activation of the C–H bond dissociation, whereas the decrease in the CO_ad_ formation rate upon deuterium substitution implies a primary kinetic H/D isotope effect (*k*_H_/*k*_D_). The observation of a kinetic isotope effect means that the C–H bond splitting appears in the rate determining step, which is either the first or the second hydrogen split-off. Previous theoretical work predicted a nearly barrier free spontaneous subtraction of the first hydrogen from adsorbed formaldehyde and a somewhat higher energy barrier for the dehydrogenation of the resulting adsorbed formyl [73]. This agrees with the experimental finding of adsorbed formyl species in the present work, since a fast dissociation of the second hydrogen would lead to a negligible coverage of the adsorbed intermediate.

The experimental *k*_H_/*k*_D_ values, which are plotted in Figure 4b, range around 1.9 ± 0.4 for 23 °C and around 2.8 ± 0.5 for 3 °C, with slightly lower values at lower potentials. Importantly, the change of the selectivity in the dehydrogenation reaction from complete dehydrogenation (to CO_ad_) at low potentials to incomplete dehydrogenation (to formic acid) at higher potentials does not induce any significant change in the *k*_H_/*k*_D_ values, indicating that in both cases the C–H bond dissociation appears in the rate determining step.

The higher value of the kinetic H/D effect at lower temperature indicates a larger slow down of the C–D dissociation vs that for the C–H bond, as expected for a higher activation barrier.

### Mechanistic implications

The results presented and discussed in the previous three sections lead us to the following conclusions on the interaction of formaldehyde with Pt:

1. An IR band compatible with an adsorbed formyl species, at ca. 1660 cm^−1^, was detected for the first time for formaldehyde adsorption as a band appearing at low potentials and low temperature (ca. 3 °C) in the initial stage of the adsorption (at very low CO_ad_ coverage). The assignment of this band to adsorbed formyl species is supported by the isotopic shift of the band to lower wave numbers upon D-labelling. Similarly to the adsorbed acetyl-type reaction intermediates, which we had identified during adsorption/oxidation of higher alcohols and aldehydes, the adsorbed formyl is proposed to act as precursor for CO_ad_ formation in formaldehyde adsorption, although we can not say from the present data whether this process proceeds directly or via interconversion to adsorbed hydroxymethylidyne. Although we can not detect the band assigned to adsorbed formyl at 0.4 V and higher potentials, due to the overlap with the negative band caused by displaced water (see Figure 2b–d), assumably, this pathway for CO_ad_ formation upon formaldehyde adsorption is active also at higher potentials.

2. Adsorbed formyl species are formed by dehydrogenation of adsorbed formaldehyde, according to HCHO → [HCHO]_ad_ → CHO_ad_ + H^+^ + e^−^. The other possible precursor, hydroxymethylidyne, does not interact sufficiently strong with Pt to be able to displace adsorbed *H*_upd_ at the low potentials where the adsorbed formyl species are detected (see also point 4).

3. The much lower absorption intensity and hence lower coverage of the adsorbed formyl species compared to that of the adsorbed acetyl-like species detected during adsorption/oxidation of longer chain alcohols/aldehydes is most easily understood by a lower barrier for C–H bond breaking for adsorbed formyl decomposition to CO_ad_ than that for C–C bond breaking required for adsorbed acetyl-type species, which leads to faster CO_ad_ formation and hence a lower steady-state coverage of adsorbed formyl species.

4. Oxidation of formaldehyde to formic acid on an adsorbate free Pt electrode is possible at potentials as low as 0.3 V, as evidenced by the transient observation of adsorbed bridge bonded formates in the initial stage of formaldehyde exposure to Pt. At later stages, the reaction is increasingly inhibited by CO_ad_ surface blocking. Considering that formation of active oxygen species from water is impossible at these potentials, we propose that under these conditions the reaction proceeds via dehydrogenation of adsorbed hydrated formaldehyde (methylene glycole), via OH-CH_2_-OH → [reaction intermediate]_ad_ → HCOOH + 2H^+^ + 2e^−^. This is supported by the observation that at lower potentials this reaction is inhibited, since dissociative adsorption of alcohols on Pt is well known to be inhibited by adsorbed *H*_upd_.

5. The kinetic isotope effect with *k*_H_/*k*_D_ values of 1.9 ± 0.4 at 23 °C and 2.8 ± 0.5 at 3 °C in the initial CO_ad_ formation implies that C–H bond dissociation plays an important role in the rate-determining step for this process. The resulting *k*_H_/*k*_D_ values exhibit no distinct potential dependence, despite of a pronounced potential dependence in the initial CO_ad_ formation rate in the range 0.0 to 0.4 V, indicating that the change of the dehydrogenation reaction selectivity from complete dehydrogenation (to CO_ad_) at low potentials to incomplete dehydrogenation (to formic acid) at higher potentials does not induce any significant change in the *k*_H_/*k*_D_ values. This in turn indicates that in both cases the C–H bond dissociation represents the rate determining step. The higher values of the kinetic H/D effect at lower temperature indicate a higher barrier for C–D dissociation than for C–H bond dissociation.

At present, we have no indication for pathways for formaldehyde oxidation to CO_2_ other than via re-adsorption and further oxidation of formic acid or via oxidation of CO_ad_ (indirect pathway), although such direct pathways, e.g., via an adsorbed formyl assisted interaction and reaction with adjacent H_2_O molecules, can not be ruled out. Calculations covering such scenarios are highly desirable and have been initiated.

## Conclusion

In an effort to identify possible reaction intermediates in the formaldehyde oxidation reaction on Pt and their role in the reaction, we have investigated the interaction of formaldehyde with a polycrystalline Pt film electrode in the range of low potentials, up to 0.4 V, where the oxidation of CO_ad_ formed to CO_2_ (indirect pathway) can be excluded. Employing in situ IR spectroscopy in an attenuated total reflection configuration (ATR-FTIRS) in a thin-layer flow cell, which allows for quick and efficient electrolyte exchange, the formation and disappearance of adsorbed species was monitored during spectro-electrochemical transients. The main results of measurements performed at low temperatures (3 °C) and using deuterium substitution in the C–H bond to optimize conditions for stabilizing weakly adsorbed reaction intermediates are: First, spectra obtained at low potentials indicate the existence of an adsorbed formyl intermediate, which is sufficiently strongly adsorbed to not be displaced by *H*_upd_. This is in agreement with the adsorbed acetyl type reaction intermediate observed for higher alcohols and aldehydes, where the latter species was detected and identified as reaction intermediate for CO_ad_ formation. At higher CO_ad_ coverages/potentials this species is not detected any more since it is either increasingly displaced by CO_ad_ or because the signal is overgrown by a negative band due to CO_ad_ induced displacement of water from the interface. Second, the transient formation of formic acid/adsorbed formate upon interaction of formaldehyde with adsorbate free Pt already at potentials as low as 0.3 V, where reactive oxygen formation from H_2_O can be excluded, points to a mechanism where the reaction proceeds via dehydrogenation of the hydrated form of formaldehyde (methylene glycol) at these potentials. This is supported by the observation that at lower potentials formic acid/adsorbed formate formation is inhibited, but not CO_ad_ formation, if the first reaction proceeds via adsorption of a weakly adsorbed alcohol (diol) species, which is known to be inhibited by a *H*_upd_ adlayer, while the latter starts from adsorption of a more strongly interacting aldehyde, which is possible also on a *H*_upd_ blocked surface.

## Experimental

The spectroscopy measurements were performed in a thin-layer flow cell in an attenuated total reflection (ATR) configuration described in detail in [28,32,74]. The cell was equipped with two Pt counter electrodes, located at the inlet and the outlet of the cell, respectively. A reversible hydrogen electrode (RHE) reference operated at ambient temperature was connected to the outlet of the cell via a Teflon capillary. To enable electrolyte exchange, two separate electrolyte supply bottles containing the supporting electrolyte (0.5 M HClO_4_) and 0.1 M formaldehyde solution in the same supporting electrolyte, respectively, were connected to the common inlet port. The electrolyte flow (ca. 50 µL s^−1^) was driven by the hydrostatic pressure of the supply bottles. The solutions were deareated by purging with high purity N_2_. The temperature of the electrolyte in the glass jacketed and thermally isolated electrolyte bottles was controlled by a cryostat (Huber Compatible Control CC1, filled with Cryo30 - Lauda), which was set to −10 °C. This allowed to keep the electrolyte temperature in the thermostated supply bottles at ca. −5 °C. The resulting temperature in the non-thermostated flow cell, which was continuously flushed with the electrolyte, was slightly higher, around 3 °C, due to the heat transfer from the surroundings.

The Pt-film working electrode was prepared by electroless deposition of Pt [21] on the flat side of a semi-cylindrical Si prism. The working electrode was pressed against the planar Kel-F cell body via a circular gasket (ca*.* 0.1 mm thickness, inner diameter 12 mm, exposed electrode area ca. 1 cm^2^, roughness factor ca. 5) to obtain a thin layer of electrolyte which can be effectively exchanged and allows a well defined mass transport from the inlet capillary positioned in the center of the cell body to six surrounding outlet capillaries located at the perimeter of the gasket [75]. The potential was controlled by a Pine Instruments potentiostate (Model AFRDE5).

The electrode surface was cleaned by cycling the potential between 0.06 and 1.5 V in 0.5 M HClO_4_ at 100 mV s^−1^ scan rate, until the typical features of the Pt base cyclic voltammogram (CV) [76] were reproduced. The potential was then stopped at the desired adsorption potential in the negative-going scan and the electrolyte was switched to a 0.1 M solution of formaldehyde in 0.5 M HClO_4_ for 3 min and then back to the supporting electrolyte. The supporting electrolyte was 0.5 M HClO_4_, prepared from suprapure perchloric acid (Merck) and from Millipore MilliQ-water (18.2 MΩ cm), for formaldehyde solution we used an aqueous solution of paraformaldehyde (methanol-free, 16 wt % (Alfa Aesar)) for H-labelled formaldehyde and D2-paraformaldehyde (98 D%, Isotec) for D-labelled formaldehyde, respectively. To prepare the D2-formaldehyde solution, a proper amount of D2-paraformaldehyde was first dissolved in hot (ca. 80 °C) high purity water, then cooled down and diluted by 0.5 M HClO_4_ to achieve a proper concentration.

For the ATR-FTIRS measurements we used a homemade mirror accessory within the sample chamber of a Varian 670i IR-spectrometer, equipped with a liquid nitrogen cooled mercury cadmium telluride (MCT) detector and an automated AutoPro5 polarizer set to 90° angle to transmit only surface-sensitive p-polarized radiation from the light source. The spectral resolution was set to 4 cm^−1^, the temporal resolution was 1 s per spectrum (co-adding 5 interferograms). The absorbance was calculated as A = −log(*R*/*R*_0_), with *R* representing the measured reflected intensity in the respective experiments, while *R*_0_ describes the reflected intensities in pure supporting electrolyte at the respective adsorption potential. This results in positive bands for increased absorbance.

The quantitative evaluation of the CO_ad_ formation rates upon formic acid adsorption was based on the CO_ad_ intensity – CO_ad_ coverage relation derived in potential dependent calibration measurements performed earlier, where the IR band intensity of linearly bonded CO was related to the CO_ad_ coverage determined mass spectrometrically via the partial pressure change in CO upon adsorption of CO dissolved in the electrolyte at constant potential [77]. This gives a linear relation between CO_ad_ coverage and the absorbance of linearly adsorbed CO in the coverage range from 8 to 70% of the saturation coverage [59]. Accordingly, rates for CO_ad_ formation were evaluated from the slope of the intensity increase of the linearly bonded adsorbed CO with time at coverages >8% of the saturation coverage.

## Supporting Information

File 1 CO adsorption transients and ATR-FTIR spectra at different potentials.
