# Supplementary material for: Adsorption and oxidation of formaldehyde on a polycrystalline Pt film electrode: An in situ IR spectroscopy search for adsorbed reaction intermediates
Source: Beilstein J Nanotechnol. 2014 May 30;5:747–59. doi: 10.3762/bjnano.5.87 (PMC4077456; doi:10.3762/bjnano.5.87)
Supplement: File 1 — CO adsorption transients and ATR-FTIR spectra at different potentials. [file Beilstein_J_Nanotechnol-05-747-s001.pdf]

Supporting Information

for

Adsorption and oxidation of formaldehyde on a polycrystalline Pt film electrode: An in situ IR spectroscopy search for adsorbed reaction intermediates

Zenonas Jusys* and R. Jürgen Behm

Address: Institute of Surface Chemistry and Catalysis, Ulm University, Albert-Einstein-Allee 47, D-89081 Ulm, Germany

Email: Zenonas Jusys - zenonas.jusys@uni-ulm.de

* Corresponding author

Additional experimental details

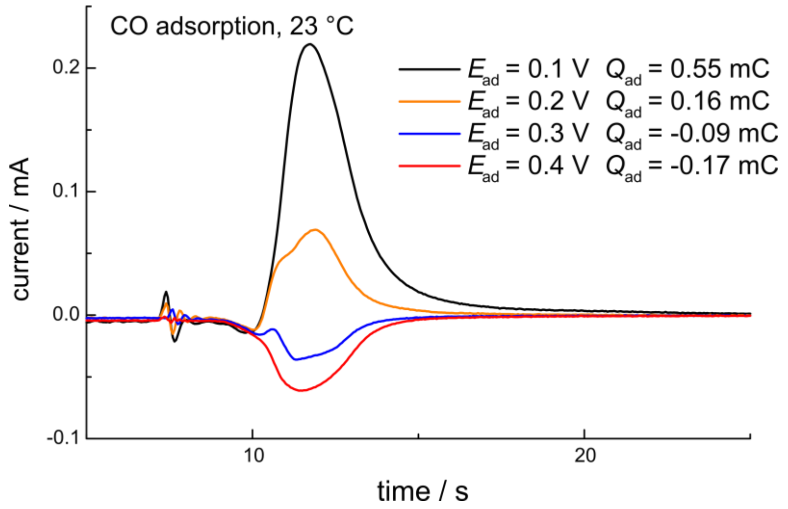

Figure S1: CO adsorption transients on a Pt film electrode at constant potentials (see Figure) upon admission of CO-saturated 0.5 M $HClO_4$ at room temperature.

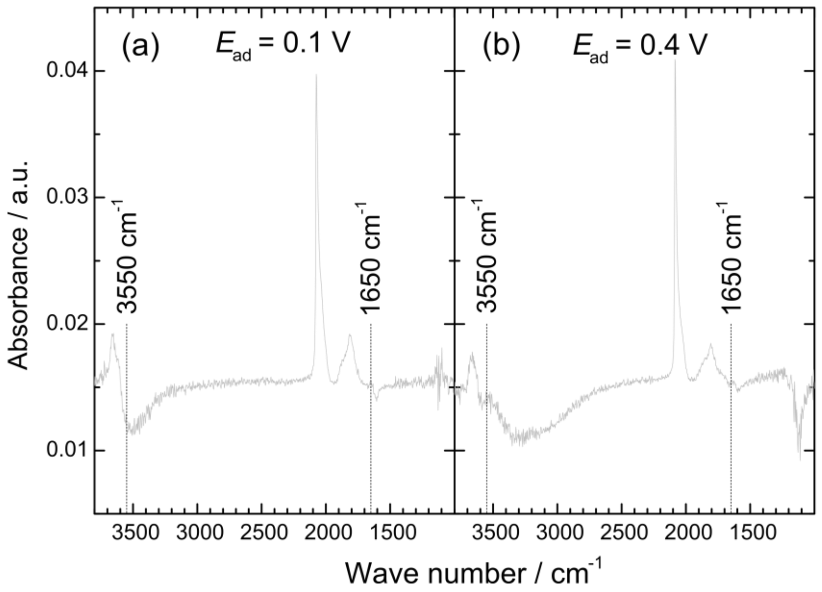

Figure S2: Initial ATR-FTIR spectra acquired ca. 2 s after admission of CO saturated 0.5 M $HClO_4$ solution to a Pt film electrode at: 0.1 V (a) and 0.4 V (b), room temperature.
